# Who Do We Remember? Facial Anomalies, Race, and Sex in Social Categorization

**DOI:** 10.3390/bs16030462

**Published:** 2026-03-20

**Authors:** Soma Chaudhuri, Isabella Bobrow, Anjan Chatterjee

**Affiliations:** 1 Penn Center for Neuroaesthetics, University of Pennsylvania, Philadelphia, PA 19104, USA; isabella.bobrow@pennmedicine.upenn.edu (I.B.); anjan@pennmedicine.upenn.edu (A.C.); 2 Department of Neurology, Perelman School of Medicine, University of Pennsylvania, Philadelphia, PA 19104, USA

**Keywords:** social categorization, group alliance, facial anomalies, race, sex, memory confusion paradigm

## Abstract

Social categorization often occurs automatically, shaping whom we notice, remember, and group together. The present study examined how visual cues indicative of sex, race, and facial anomaly guide spontaneous categorization, testing the hypothesis that anomaly-based categorization is more malleable than categorization by race or sex. Using a within-subjects Who-Said-What (WSW) paradigm, participants viewed faces that varied by sex, race, and presence of a facial scar, each paired with self-descriptive statements. A surprise recall task required matching statements to faces. Categorization strength was computed from recall errors. Participants showed the strongest categorization by sex, weak categorization by race, and very weak categorization by facial anomaly. Regression analyses revealed that scar-based categorization was negatively associated with sex- and race-based categorization. When sex or race was strongly encoded, scar-based categorization was sharply diminished, and the cue appeared only under relatively weak and infrequent conditions. Thus, although visually salient, facial anomalies did not function as an independent or stable basis for social grouping. These findings demonstrate that the categorization system prioritizes evolutionarily primary cues such as sex, treats race as a comparatively weaker cue, and assigns facial anomalies to a minimal and malleable role. Overall, the results highlight the fragile, low-priority, and easily overshadowed nature of anomaly-based categorization in social memory. Importantly, the fragility of scar-based categorization suggests that negative evaluations of anomalous faces (anomalous-is-bad stereotyping) are not automatically translated into robust memories or categorical organization.

## 1. Introduction

Categorization is a universal feature of human cognition. We all categorize, whether organizing information, classifying objects, or perceiving and judging other people. It is how we make sense of a complex world. Social categorization refers to the process by which people categorize themselves and others into differentiated groups with whom they might ally, and serves as a heuristic to navigate the social world (Krueger, 2001). The grouping process is typically automatic and spontaneous (Weisman et al., 2015), It guides how we learn, perceive, interpret social information, and ultimately behave toward others (Turner et al., 1994; Lickel et al., 2000; see Rhodes & Baron, 2019).

Human faces carry rich visual information that people use to infer others’ traits and group memberships, making facial appearance a central basis for social perception and categorization (Macrae & Quadflieg, 2010). People automatically categorize others into social groups upon seeing their faces (Macrae & Martin, 2007). Decades of research indicate that race, sex, and age are the “Big Three” social categories that people use as traditional cues to categorize others (Zhao & Bentin, 2008; see Rule & Sutherland, 2017) (Messick & Mackie, 1989; Fiske & Neuberg, 1990). This categorization occurs in a largely automatic and obligatory manner in the absence of any individuating information (Fiske & Neuberg, 1990; Taylor et al., 1978; Hewstone et al., 1991; Stangor et al., 1992; see Kurzban et al., 2001). However, not all informational cues appear equally robust. From an evolutionary perspective, Kurzban et al. (2001) argued that sex and age function as evolved automatic encoding mechanisms, whereas race does not, since ancestral humans rarely encountered individuals from racially distant populations. They proposed that no part of the human cognitive architecture is specifically designed to encode race; instead, what appears to be automatic racial encoding is a byproduct of cognitive systems that evolved to track social alliances and group membership. Conversely, a related line of evidence shows that social categories are activated unintentionally upon seeing a face. For example, in a study, Macrae and Martin (2007) showed that participants identified the gender associated with a name more quickly when they first saw a face of that gender, than if they instead saw the face of a person of the opposite gender, indicating automatic gender categorization (Rule et al., 2009; see Rule & Sutherland, 2017). However, empirical evidence further indicates that people can identify social categories even when the perceptual distinctions are ambiguous, for example, inferring sexual orientation or political affiliation from faces at above-chance levels (with accuracy rate of about 65%) (Tskhay & Rule, 2012), although to a lesser degree than for perceptually obvious categories (Macrae & Quadflieg, 2010; see Rule & Sutherland, 2017).

Although social categorization simplifies perception, it also brings potential downstream consequences: it can support biased beliefs, including unwarranted stereotypes (Allport et al., 1954; see Liberman et al., 2017). A stereotype refers to “an individual’s set of beliefs about the characteristics or attributes of a group” (Judd & Park, 1993). Early theories (Allport et al., 1954) proposed stereotyping of racial and ethnic groups as intrinsic to the cognitive system (see Taylor et al., 1978). In social interactions, physical appearance, along with sexual identity, represents salient personal characteristics. The “beauty-is-good” stereotype is a well-established finding in social psychology, describing the attribution of positive characteristics to physically attractive individuals (Dion et al., 1972). This stereotype reveals that physically attractive individuals are consistently attributed with more positive traits and socially desirable personalities than their less attractive counterparts (Dion et al., 1972). Regardless of the rater’s sex or that of the target, attractive people are perceived as more likable, sociable, and competent, illustrating the pervasive influence of physical appearance on social evaluation (Dion et al., 1972).

In contrast, visible facial differences often evoke opposite impressions. Complementing the “beauty-is-good” stereotype, the “anomalous-is-bad” stereotype suggests that individuals with visible facial differences are perceived not only as less attractive but also as less moral and possessing more negative personal characteristics (Hartung et al., 2019; Jamrozik et al., 2019; Workman et al., 2021). People with visible facial anomalies, such as scars or palsies, are often judged as less warm and less competent, reflecting the ‘anomalous-is-bad’ stereotype. This bias appears regardless of whether the anomaly is internal (e.g., palsy) or externally caused (e.g., scars) (Paruzel-Czachura et al., 2024). Some prior research links these negative impressions to pathogen-avoidance motives, since anomalies may resemble cues historically associated with infectious disease or illness (Ryan et al., 2012; Oaten et al., 2011; Paruzel-Czachura et al., 2025). Of note, recent evidence questions the universality of this proposition (Tybur et al., 2022; Workman et al., 2022). Given claims about evolutionary mechanisms of pathogen sensitivity, it remains unclear whether facial anomalies themselves are salient enough to function as a salient cue for spontaneous social categorization, and how far this bias extends into social perception.

### The Present Study

The present study examined how visual cues to sex, race, and facial anomalies drive spontaneous social categorization when individuals are simply observed rather than explicitly judged. We used the “Who Said What?” (WSW) paradigm (Taylor et al., 1978), a memory-confusion method to measure spontaneous social categorization from recall errors. This paradigm has been widely adopted in social cognition research (Lieberman et al., 2008; van Leeuwen et al., 2012; Bor, 2017; Kurzban et al., 2001; Pietraszewski & Schwartz, 2014; Pietraszewski, 2021; Pietraszewski et al., 2014). Recent work by Flade and Imhoff (2024) helps explain this pattern: the WSW task is a source-memory paradigm in which participants view each face multiple times and must remember not only the face but also the statement associated with it. This makes the task more demanding than other-race face recognition tasks (Guo et al., 2021; Oruc et al., 2019; see Flade & Imhoff, 2024). One advantage of this method is that “it allows us to see how subjects spontaneously view this social world” (Delton & Robertson, 2012, p. 718; see Bor, 2018). In the WSW paradigm, participants view several faces, each delivering several statements. Participants later complete a recall task identifying which person said each statement. Categorization is revealed from misattributions: if a statement made by one target is incorrectly attributed to another who shares the same feature (e.g., a Black speaker mistaken for another Black speaker), this indicates that the observer grouped those individuals together based on the cue to race. A higher proportion of within-category errors therefore reflects stronger spontaneous categorization along that cue (Taylor et al., 1978; see also Bor, 2018).

Using this approach, the present study examined whether facial anomalies organize spontaneous categorization, and how their influence compares with well-studied dimensions of race and sex. Scars serve as the focal anomaly, selected for their visual salience and prior use in research on the “anomalous-is-bad” stereotype (Workman et al., 2021; Paruzel-Czachura et al., 2024). Specifically, we selected healed burn scars as the anomaly cue because they are visually salient and easily identifiable. In this pre-registered study (https://doi.org/10.17605/OSF.IO/2SPGF), we formulated one hypothesis accompanied by two predictions:

**Hypothesis:**  *Facial anomalies as a source of social categorization are malleable compared to race and sex.*

Prediction 1: Facial anomaly-based social categorization is modulated by race and sex cues. Participants will be less likely to encode based on facial anomalies when race and sex cues compete. That is, scar-based categorization decreases when race/sex categorization is robust.

Prediction 2. Scar-based categorization will only emerge when sex and race-based categorization are weak. This weakening of traditional cues was expected to be greater for sex than for race.

These predictions concern how scar-based categorization relates to the use of dominant social cues, focusing on both how scar-based categorization changes as race and sex cues become more robust and the conditions under which scar-based categorization is expected to be expressed. Although closely related, these predictions address complementary questions: Prediction 1 concerns whether scar-based categorization is systematically modulated by race and sex cue strength (tested via the overall regression model), whereas Prediction 2 concerns whether this modulation creates conditions under which scar-based categorization can function as a meaningful organizing dimension (tested via conditional patterns in simple slopes analysis).

## 2. Materials and Methods

### 2.1. Materials

All face-stimuli were sourced from the Face Research Lab London Set (DeBruine & Jones, 2017). The database specifies that the individuals photographed provided consent for their images to be used for scientific research, including in altered form and in published materials. To create scarred versions of these faces, we used AI-based image generation tools (via ChatGPT, GPT-5) to simulate realistic, plausibly healed facial burn scars. Scar placement varied across faces (e.g., on the left or right cheek) to prevent location-based confounds and to enhance ecological validity.

We created a corpus of 24 brief, first-person statements describing everyday activities (e.g., hobbies, food, routines, pets), written in the present tense. These statements were designed to be short, with an average length of 5.33 words (*SD* = 1.40), minimizing variability in sentence complexity and reducing potential confounds related to cognitive load or memory demands. To further control for content-related bias, the statements were sentiment-balanced (18 were neutral and 6 were mildly positive) with a low overall mean polarity of 0.055 (*SD* = 0.094). Sentiment polarity scores were computed using the *sentimentr* package in R. The selection of statements ensured that differences in recall or categorization could not be attributed to emotional tone or semantic bias in the verbal stimuli. Example statements included “I read poetry,” “I read the newspaper daily,” and “I have a pet dog,” among similar everyday items.

All face stimuli, statement metadata, and the full encoding matrix (face–statement pairings) are publicly available on OSF: https://osf.io/kysne/overview?view_only=5a70a1e22c144cd39340b5a c5d688a74 (accessed on 17 March 2026).

### 2.2. Participants

We recruited a total of 500 participants between the ages of 18 and 65 years from the United States via Prolific to complete an online survey administered through Qualtrics. We used attention checks within the encoding block to ensure engagement. As specified in the preregistration (https://doi.org/10.17605/OSF.IO/2SPGF), participants who failed all three checks were specified for exclusion. In the present sample, no participants met this exclusion criterion. Additionally, as a self-reported data-quality check (Curran, 2016), participants were asked at the end of the study whether they believed their data was high-quality enough to be included in analysis. Three participants responded negatively and were removed according to preregistered exclusion criteria, yielding a final sample of 497 (Mean age = 41.53 years, *SD* = 11.11; male = 247, female = 245, intersex = 2, prefer not to say = 3). The survey took approximately 15 min, and participants were compensated $4 for their time.

### 2.3. Procedure

**Figure 1:**
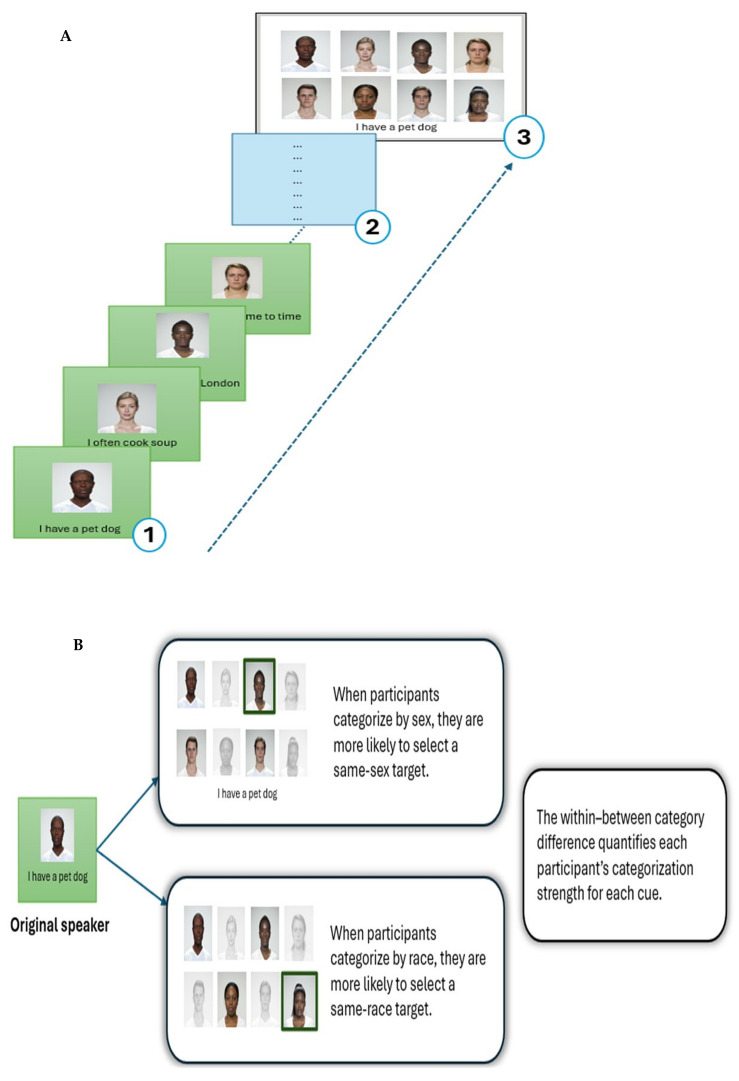
Schematic of the memory confusion paradigm used to measure social categorization. (**A**). During encoding, participants viewed a sequence of faces paired with statements (1), followed by a brief distractor task to prevent rehearsal (2). In the subsequent surprise memory test (3), participants selected the face they believed had made each statement, presented in random order. (**B**). An example illustrating the categorization logic. Participants who categorized face stimuli by sex or race were more likely to select same-sex or same-race targets, respectively. The within–between-category difference quantifies each participant’s categorization strength for each cue, such that participants who categorized by a given cue (e.g., sex, race, or facial anomaly) were more likely to confuse individuals within that same category.

The preregistered study (https://doi.org/10.17605/OSF.IO/2SPGF) employed a within-subjects design based on the Who-Said-What? paradigm. The procedure comprised three distinct phases: (1) an encoding phase, (2) a short distractor task, and (3) a surprise memory recall task (see Figure 1).

**Figure 2:**
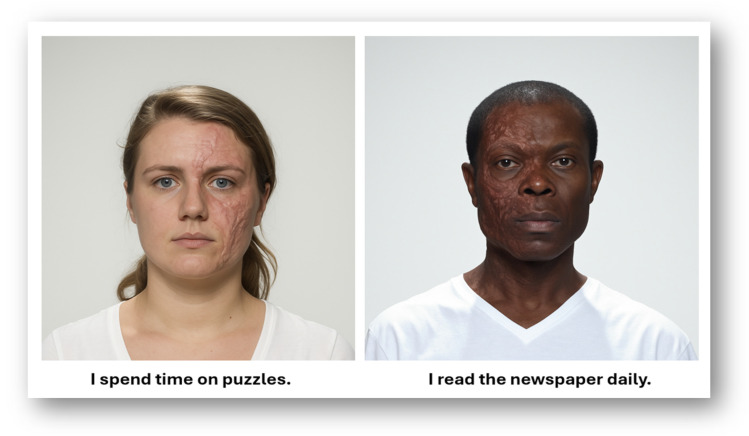
Two scar-present faces are shown here, each paired with one of the 24 self-descriptive statements used in the study. The full stimulus set included eight faces (crossed by sex, race, and scar presence). Although displayed side by side in this figure for clarity, all stimuli were presented one at a time and in randomized order during the encoding phase. Source: (DeBruine & Jones, 2017; https://doi.org/10.6084/m9.figshare.5047666.v5).

During the encoding phase, participants viewed eight unique faces, balanced across race (Black/White), sex (male/female), and facial anomaly (with scar/without scar). Each face was paired with one of three brief, first-person statements describing everyday experiences (see Figure 2 for examples). Consequently, there were 24 unique face–statement pairings, presented in randomized order. Each trial was displayed for 10 s, and participants were instructed to view each pairing carefully. They were not informed that a memory test would follow, ensuring that later recall reflected spontaneous encoding processes rather than intentional memorization. Of note, to ensure attentiveness, three randomized attention checks were embedded within the encoding phase. Participants who failed all three attention checks were to be excluded from analyses, in accordance with the pre-registered exclusion criteria.

Immediately after the encoding block, participants completed a short distractor task designed to prevent short-term rehearsal and to reduce recency effects. A stem-completion task using U.S. city names was used for this purpose (Mulligan & Hartman, 1996). Participants were instructed to complete each city name stem by typing a valid U.S. city beginning with the given letters (e.g., Chi_ → Chicago). The task was unrelated to the main experimental procedure and served to mitigate short-term memory carryover effects between encoding and recall phases. Participants were asked to complete as many items as possible within the allotted time and were informed that there were no right or wrong answers.

After the distractor task, participants completed a surprise memory recall task. Each of the 24 statements was presented randomly, one at a time, accompanied by a constant grid displaying all eight faces. For each statement, participants were instructed to select the face they believed had originally made that statement. They were asked to do their best, with no mention of correct or incorrect responses. The recall task was self-paced, allowing participants to engage in natural memory retrieval and minimizing pressure-related variability, thereby enhancing ecological validity. While the order of statements was randomized across participants, the face grid remained constant throughout the task to ensure clear within-subject comparability in categorization patterns. Figure 1 illustrates the experimental procedure based on the memory confusion paradigm.

After completing the three experimental phases, participants responded to a brief questionnaire about their sociodemographic characteristics (e.g., age, gender, race/ethnicity) and exposure to individuals with facial scars. The exposure-related questions were designed to capture both the frequency and personal relevance of participants’ experiences with facial anomalies. Frequency of exposure was measured with the item, “How often do you encounter individuals with visible facial scars in your daily life?” (1 = Never to 5 = Regularly). Personal acquaintance was assessed with the question, “Do you personally know someone with a visible facial scar?” (response options: family member, coworker, or none). Self-scar status was measured with the item, “Do you yourself have a visible scar or disfigurement?” (Yes/No). These variables collectively indexed both the extent and personal salience of participants’ exposure to facial anomalies. As these analyses were exploratory, no specific directional hypotheses were proposed; rather, they aimed to examine whether greater or more personal exposure corresponded to weaker scar-based categorization biases.

### 2.4. Analysis

Analysis focused on participants’ errors made in the memory recall task. Correct responses, where participants selected the true speaker, were ignored, because a correct match could arise from accurate memory, reliance on category information, guessing, or a combination of these processes, making them uninformative for assessing categorization strength (Pietraszewski et al., 2014; Bor, 2018). Therefore, analyses were restricted to incorrect trial only, because these trials permit categorization in the WSW paradigm (Pietraszewski et al., 2014). Errors made by participants could be of two types: within-category errors and between-category errors. A within-category error indicates that a participant used that feature to group the targets, recognizing that the statement came from someone in the same category but mis-remembering the specific person. For example, confusing (mistaking) one man for another man reflects reliance on sex as a grouping cue. In contrast, a between-category error, such as mistaking a man for a woman, provides no evidence that the sex was used for grouping.

To establish that errors reflected non-random responding rather than indiscriminate guessing, we examined whether memory confusions tended to share at least one social feature (sex, race, or scar) with the true speaker. In a 2 × 2 × 2 design, the probability that two randomly selected faces share at least one feature is 6/7 (≈85.7%). On average, participants made errors in which 91.9% of chosen faces shared at least one feature with the true speaker (*SD* = 10.1%), which was modestly but reliably above the chance level, *t* (485) = 13.61, *p* < 0.001, *d* = 0.62. At the trial level, 4593 of 5143 errors (89.3%) involved feature overlap, which was also modestly but reliably above the 85.7% baseline (exact binomial test, *p* < 0.001). This indicates that memory confusions were not randomly distributed across faces. Given the reliable deviation from random guessing, no base-rate correction was applied (Bor, 2018).

To quantify categorization by each cue, a difference score is calculated for each subject. Following standard practice (Pietraszewski et al., 2014), categorization strength was operationalized as the difference between within-category errors (mistaking a target for another person who shared the same feature, e.g., same race) and between-category errors (mistaking a target for someone from a different feature category).

For each visual cue—race, sex, and facial anomaly—a proportional categorization score was computed using the conventional formula:

$$\mathrm{Categorization}\mathrm{score}\left(\mathrm{proportional}\right)=\frac{\mathrm{within}\mathrm{category}\mathrm{error}-\mathrm{between}\mathrm{category}\mathrm{error}}{\mathrm{total}\mathrm{errors}}$$

Categorization scores were computed following standard Who-Said-What procedures. Each score reflects the proportion of all incorrect responses in which the chosen face shared the relevant category with the true speaker, irrespective of whether other dimensions (e.g., race or scar) also matched. Categorization scores were therefore not mutually exclusive, consistent with the assumption that multiple social dimensions can be encoded simultaneously. Higher values indicate stronger categorization based on that feature. These scores served as the categorization indices for subsequent analyses.

A trial-level GLMM was preregistered to analyze binary outcomes. However, when implemented, this model proved statistically unidentifiable due to structural properties of the error distribution, including the absence of some cue combinations. The resulting parameter estimates were unstable and not interpretable. Accordingly, and consistent with reports in the Who-Said-What literature (Taylor et al., 1978; Kurzban et al., 2001; Pietraszewski et al., 2014), we report analyses based on participant-level categorization indices, which directly address the research question.

To test whether scar-based categorization varied as a function of race- and sex-based categorization, we conducted a multiple regression analysis in which scar-based categorization scores were predicted from race- and sex-based categorization and their interaction. This model assessed whether the extent to which participants categorized people by race and sex systematically shaped their tendency to categorize by facial anomaly.

Because categorization scores in the Who-Said-What paradigm are derived from a finite pool of memory errors, scores for different cues are not statistically independent and may exhibit mechanical tradeoffs. To evaluate whether the observed regression results could arise from these shared-error constraints alone, we conducted a null simulation preserving each participant’s number of errors and the 8-face task structure.

On each error trial, the chosen face was randomly sampled from the 7 incorrect alternatives, thereby retaining all zero-sum constraints while eliminating any meaningful cue-based encoding. This approach isolates the contribution of measurement artifacts from genuine psychological processes: any relationships emerging in the null data reflect mathematical constraints alone, whereas relationships exceeding the null distribution reflect structured cue-based categorization. Categorization scores for sex, race, and scar were recomputed from the simulated data using the same scoring procedure as in the primary analysis, and the regression model was refit 5000 times. The resulting distributions of explained variance and regression coefficients were used to assess whether the observed effects exceeded those expected under mathematical constraints alone. The regression model was used to evaluate both the degree to which scar-based categorization decreases as race- and sex-based categorization become more robust (Prediction 1) and whether scar-based categorization is evident specifically under weak race- and sex-based categorization (Prediction 2).

Further, we explored whether real-world exposure to facial anomalies relates to how strongly participants categorized by scars. For this, we fit a regression model including race- and sex-based categorization and their interaction. We then expanded this model by incorporating participants’ real-world exposure indicators: how often they encounter individuals with visible facial scars, whether they personally know someone with a scar, and whether they themselves have one. This analysis allowed us to test whether exposure, as reported subjectively, contributes meaningfully to variation in anomaly-based encoding beyond the primary social cues. Finally, because older people may have had greater lifetime exposure to facial anomalies, a regression model tested whether age moderated the association between exposure frequency and scar-based categorization. For completeness, the model was also re-estimated in a subsample restricted to participants aged 30 and above, since younger adults may have had relatively limited lifetime exposure opportunities. Together, these exploratory analyses were intended to examine whether individual experience and demographic factors shape the extent to which people use facial anomaly cues during spontaneous social categorization.

## 3. Results

### 3.1. Descriptive and Inferential Statistics

**Figure 3:**
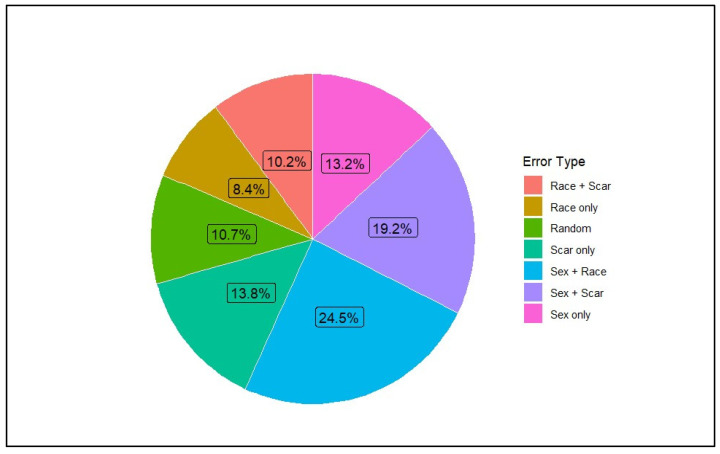
Distribution of all incorrect memory-attribution errors (*n* = 5143) across cue types in the Who-Said-What task. Each segment reflects the proportion of confusions in which participants misattributed a statement to a person sharing a specific cue (e.g., sex only, race only, scar only) or a combination of cues. The “Random” category reflects errors in which no cue matched between the chosen and actual speaker. This distribution illustrates the trial-level frequency of different cue combinations present in memory errors. Note. Categories are not mutually exclusive; errors can involve multiple cue matches (e.g., Sex + Race). Categorization scores were computed using all errors sharing the relevant cue, not only single-cue-only errors.

The proportion of all incorrect responses (*n* = 5143) that involved each cue or cue combination is illustrated in Figure 3. Descriptive statistics for categorization scores are shown in Table 1. Categorization by sex was robust (*M* = 0.29, *SD* = 0.44, *t* (485) = 14.63, *p* < 0.001, *d* = 0.66), indicating strong and consistent reliance on sex as a grouping cue. Race-based categorization was minimal (*M* = −0.04, *SD* = 0.48, *t* (485) = −2.05, *p* = 0.041, *d* = 0.09), suggesting a small effect and suggesting negligible reliance on racial information. Scar-based categorization was the weakest of the three cues (*M* = −0.17, *SD* = 0.44, *t* (485) = −8.43, *p* < 0.001, *d* = −0.38), indicating that participants were less likely to confuse individuals who shared the same scar status, suggesting that scars did not function as a meaningful grouping cue. Of note, a negative score reflects fewer within-category errors than between-category errors for scars, suggesting that facial anomalies did not operate as a grouping cue and may instead elicit a contrast or avoidance pattern rather than meaningful categorization.

**Figure 4:**
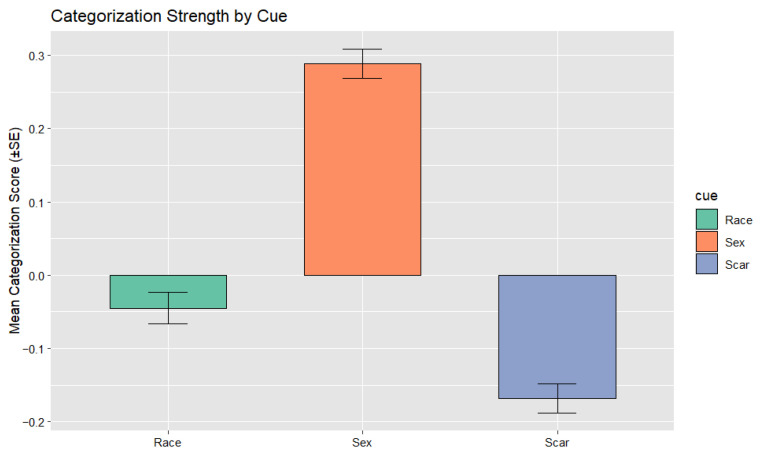
Mean categorization strength (±SE) as a function of cue type. Bars represent participant-level average proportional categorization scores, computed as (within − between)/total errors, for race, sex, and facial anomaly (scar). Error bars show ±1 SE of the mean. Positive values indicate a greater tendency to confuse individuals within the same category, reflecting stronger spontaneous categorization; negative values indicate fewer same-category confusions than different-category confusions. Participants showed robust categorization by sex (*M* = 0.29), minimal categorization by race (*M* = −0.04), and negative categorization for scars (*M* = −0.17), indicating that facial anomalies were the weakest systematic organizing dimension.

Effect-size magnitudes showed a clear hierarchy: sex categorization was strong, race categorization was small in magnitude, and scar categorization was small-to-medium in the opposite direction (see Cohen, 1988). This pattern confirms that sex was the most strongly encoded cue, followed by minimal race-based categorization and a negative scar-based pattern. Figure 4 shows the relative strength of categorization across cues.

### 3.2. Cue Interdependence

Although scar-based categorization was the weakest overall, indicating that scars did not function as a general grouping cue, it remained possible that anomaly-based encoding might only emerge in specific conditions: when more familiar cues like sex and race were not driving memory organization, albeit a rare occurrence. In other words, if participants rely on scars at all, such reliance may appear primarily among those who do not strongly encode sex or race. If so, scar-based categorization would not appear as a population-level tendency but would emerge primarily among people who encode sex or race less strongly.

To test this possibility, we examined whether reliance on the familiar cues (race and sex) predicted the extent to which participants relied on facial anomalies. We estimated a multiple regression model in which scar-based categorization scores were regressed onto race-based categorization, sex-based categorization, and their interaction. The regression model was significant, *F* (3, 482) = 181.4, *p* < 0.001, accounting for 53% of the variance in scar-based categorization (*R*^2^ = 0.53) (see Table 2). Both race-based categorization (*b* = −0.32, *p* < 0.001) and sex-based categorization (*b* = −0.15, *p* < 0.001) were significant negative predictors of scar-based categorization. These inverse associations are consistent with our prediction that anomaly-based categorization is relatively weak and tends to be lower among participants who show stronger categorization by sex and race.

**Figure 5:**
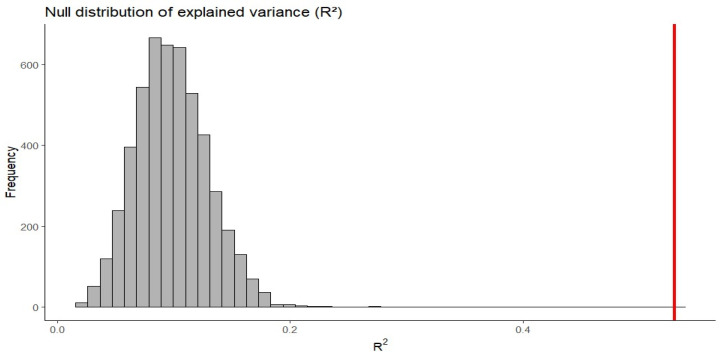
Null distribution of explained variance (*R*^2^). The histogram shows *R*^2^ values from 5000 null simulations preserving each participant’s number of errors and the task structure while randomizing face selection on error trials. The red vertical line indicates the observed *R*^2^ (0.53), which exceeded all simulated values (99.9% quantile = 0.21, *p* < 0.001), demonstrating that the regression model likely captures genuine cue-based categorization beyond mathematical constraints alone.

To assess whether these relationships could arise from shared-error constraints alone, we conducted a null simulation. The observed *R*^2^ (0.53) exceeded all values obtained under the null (99.9% quantile = 0.21, *p* < 0.001; see Figure 5), indicating that the regression model captures genuine cue-based encoding processes beyond what mathematical constraints alone would produce. The Sex × Race interaction coefficient (*b* = −0.51) lay in the tail of the null distribution (*p* = 0.023; see Figure S1). Specifically, 2.3% of null simulations produced interaction coefficients of comparable magnitude or greater. However, this occurred in the context of an overall model fit far exceeding chance. While shared-error constraints contribute some mechanical tradeoff among categorization scores, the magnitude of negative association observed substantially exceeds baseline expectations and reflects structured relationships among participant-level categorization strengths, with scar-based categorization being lowest when primary cues are strongly encoded. In this context, we use the term “primary cue” to refer to a dimension that consistently produces stronger within-category than between-category confusions across participants, rather than to the frequency of specific single-cue error types.

**Figure 6:**
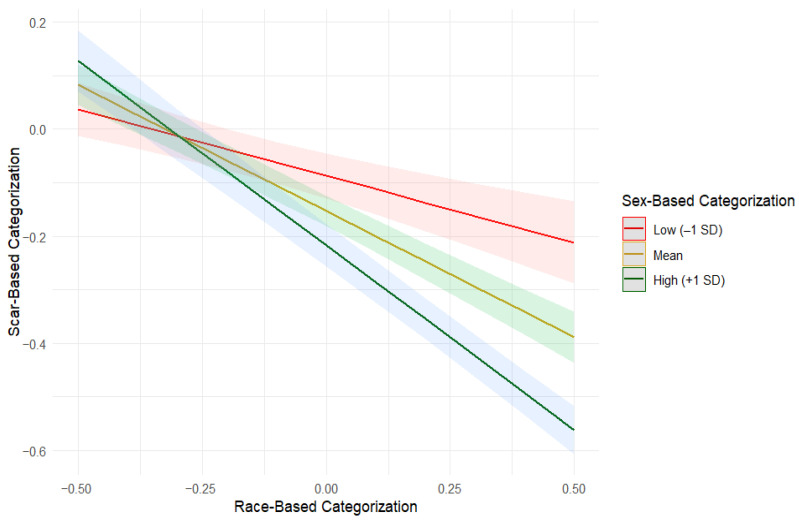
Interaction between race-based and sex-based categorization predicting scar-based categorization. Predicted slopes are shown at low (−1 SD), mean, and high (+1 SD) levels of sex-based categorization. Shaded areas indicate 95% confidence intervals. The interaction illustrates that scar-based categorization becomes weaker as race-based categorization increases, with this decline being most pronounced when sex-based categorization is strongly used.

The model also revealed a significant Race × Sex interaction (*b* = −0.51, *SE* = 0.06, *t* = −8.16, *p* < 0.001), indicating that scar-based categorization was lowest among participants who showed strong categorization by both sex and race. That is, anomaly-based encoding was weakest when the more dominant social cues were strongly used. Simple-slopes analyses (Table 3, Figure 6) further illustrated this pattern: even across race’s uniformly weak range, relatively higher race-based categorization predicted lower scar-based categorization. This decline was substantially steeper when sex-based categorization was strong compared to when it was at the lower end of its distribution (though an infrequent occurrence in our data). The difference between these slopes was significant (slope difference = 0.44, *SE* = 0.05, *t* = 8.16, *p* < 0.001).

These findings support Prediction 1 but provide limited support for Prediction 2. Scar-based categorization decreased as race- and sex-based categorization increased, with this decline most pronounced when both cues were strongly encoded (Table 3). However, scar-based categorization remained near zero or negative even when race and sex encoding were at their weakest observed levels.

### 3.3. Exposure and Age Effects on Scar-Based Categorization

To examine whether familiarity with facial anomalies influences the extent of scar-based categorization, we conducted an exploratory multiple regression model that included exposure frequency, acquaintance type, and self-scar status in addition to the core categorization predictors (race-based categorization, sex-based categorization, and their interaction). The key question was whether exposure-related factors contributed anything above and beyond the core scar-based categorization dynamics. The overall model was significant, *F* (7, 473) = 77.93, *p* < 0.001, explaining 53.6% of the variance in scar-based categorization (*R*^2^ = 0.536). However, this significance was driven entirely by the race- and sex-based predictors, which remained strong and highly significant (all *p*s < 0.001), including their interaction. None of the exposure-related variables significantly predicted scar-based categorization. Exposure frequency showed a small nonsignificant negative trend (*b* = −0.04, *p* = 0.096), consistent with the possibility that more frequent encounters with facial anomalies might slightly reduce scar-based categorization. Acquaintance type (coworker or family) and self-scar status were wholly unrelated to scar-based categorization (all *p*s > 0.10). A model comparison further confirmed that adding these exposure variables did not improve model fit beyond race and sex cues alone, Δ*F* (4, 473) = 1.34, *p* = 0.25, Δ*R*^2^ = 0.011. Thus, there is no reliable evidence that exposure frequency or personal familiarity alters reliance on scar cues (see Table S1).

Given that older adults may have had greater real-world exposure to people with facial anomalies, and because exposure frequency showed a small but nonsignificant trend, we tested whether age moderated the relationship between exposure frequency and scar-based categorization. A linear regression including Age, Exposure Frequency, and their interaction revealed no significant effects, *F* (3, 482) = 0.76, *p* = 0.52, *R*^2^ = 0.005, and neither the main effect of age nor the interaction term approached significance (both *p*s > 0.20) (See Table S2). Further, to ensure this null effect was not obscured by younger participants with low exposure, the analysis was repeated in a restricted sample of participants aged 30 and above (*n* = 412). Results were unchanged; the model remained nonsignificant, *F* (3, 408) = 1.71, *p* > 0.10, indicating that age did not moderate exposure frequency at either sample level. Thus, there was no evidence that anomaly-based categorization changes with age or accumulated exposure experience, which suggests that scar-related encoding may reflect a stable perceptual process rather than one shaped by social contact. Regression results and exploratory visualizations of exposure and age effects are presented in the Supplementary Materials (Tables S1 and S2 and Figure S2).

## 4. Discussion

Social categorization is a fundamental process in everyday interactions. People spontaneously categorize others into meaningful groups based on socially informative features. Social psychology research consistently identifies sex and race as important cues guiding spontaneous social categorization and establishing group alliances (Zhao & Bentin, 2008). Meanwhile, growing work in neuroaesthetics demonstrates the “anomalous-is-bad” stereotype, showing that individuals with visible facial anomalies are often judged as less moral or socially desirable (Workman et al., 2021). What remains unknown is whether anomalies act not only as evaluative signals but also to structure social categorization. Do facial anomalies guide spontaneous, non-evaluative grouping in the same way that race or sex do? The present study tested this possibility using the Who-Said-What memory-confusion paradigm (Taylor et al., 1978), which measures spontaneous categorization from within-category memory errors by tracking whether participants confuse individuals who share the same cue (e.g., features suggestive of race, sex, or facial anomaly). A higher proportion of within-category confusions reflects stronger automatic grouping along a cue. We found that categorization was strongest for sex, minimal for race, and weakest for facial anomaly. Scars did not function as a stable or primary basis for grouping and anomaly-based categorization appeared relatively fragile and weaker than categorization based on familiar social cues.

Understanding whom we remember, and how these memory processes guide the way we categorize others, is fundamental in social cognition. Facial appearance is one of the most immediately accessible social cues (Dion et al., 1972). Among the “Big Three” cues—sex, race, and age—sex and age are considered evolutionarily primary, whereas race is thought to reflect a more flexible, coalition-tracking mechanism rather than a hardwired category (Stangor et al., 1992; Kurzban et al., 2001). Stereotyping reflects core cognitive processes that organize social information (Allport et al., 1954). Individuals with facial anomalies are consistently perceived as less warm, less competent, or less socially desirable, reflecting the “anomalous-is-bad” stereotype documented in neuroaesthetics research (Workman et al., 2021; Paruzel-Czachura et al., 2024). Consistent with evolutionary accounts, people tend to view facial normalcy or typicality as markers of beauty, whereas facial disruptions or anomalies are often perceived as cues to infectious disease. Thus, even when they are not often linked to any actual illness, facial anomalies may still be interpreted as signals of pathogen threat and may activate pathogen-avoidance mechanisms in perceivers (Zebrowitz & Montepare, 2008; Tybur et al., 2009).

If facial anomalies do indeed tap into evolutionarily rooted disease-avoidance mechanisms, they might function not only as evaluative cues but also as social sorting cues, shaping how people spontaneously group others during memory and perception. This possibility could extend the “anomalous-is-bad” stereotype’s broader social implications. However, emerging cross-cultural evidence challenges the universality of this “anomalous-is-bad” response. Workman et al. (2022) argue that the “anomalous-is-bad” stereotype might not be shaped by cultural factors and not a signal linked to historic pathogen risk mechanisms (Schaller & Park, 2011). These possibilities raise open questions central to the present study: Do facial anomalies operate as automatic categorization cues, like traditional cues like sex or race? Did such categorization evolve or are they culturally learned and thus more malleable as socially meaningful signals? We address this question directly by examining whether facial anomalies guide spontaneous social categorization in a neutral, non-evaluative context, and whether they do so alongside or in competition with traditional social cues.

Using the classic Who-Said-What paradigm (Taylor et al., 1978), the present study found clear differences in categorization strength across three visually salient cues: sex, race, and facial anomaly. Sex produced the strongest categorization, race showed minimal, and facial anomaly showed a weak categorization effect overall. Scar-based categorization was lowest in the region of the data where both sex- and race-based categorization were strong—the most common pattern in the sample. The only context in which scar-based grouping was noticeable was when race-based categorization was relatively weak and sex-based categorization was strong; however, this combination was comparatively uncommon (about 16.5% of participants, identified via median-split quadrant analysis; see Supplementary Table S3). Thus, although this pattern is theoretically informative, it occurred infrequently and did not reflect a general tendency. Overall, scars were not a stable or primary basis for grouping. Instead, anomaly-based categorization was relatively weak compared with familiar social cues and tended to be higher only in specific and relatively rare cue-combination profiles.

These patterns suggest that the mind does not treat facial anomalies as a fundamental way of carving up the social world. Instead, scars are folded into an existing hierarchy of cues. Sex remained the dominant organizing dimension, race contributed only modest structure, and scars did not shape grouping on their own. When sex and race offer a clear way to sort people, which is most common in social interactions, people do not typically emphasize facial anomalies in remembering others for mental grouping. Only when the traditional cues, particularly race, show the lowest encoding, scars are statistically noticeable as a relevant cue for grouping. Interestingly, this pattern occurred infrequently across the sample, which is quite relevant in everyday life. This apparent asymmetry aligns with evolutionary accounts of social perception. Sex and age mark ancestrally important dimensions for reproduction, cooperation, and competition, and are thought to be supported by specialized, robust encoding mechanisms. Race, by contrast, probably reflects a flexible alliance-tracking system rather than a dedicated, hardwired ancestral category (Kurzban et al., 2001). If facial anomalies genuinely tapped into a deeply rooted pathogen-avoidance system, we might expect them to function as a strong, automatic basis for categorization across contexts similar to sex. Our findings do not support this possibility. Despite their visual salience and their clear role in negative evaluation (the “anomalous-is-bad” stereotype), scars did not behave like a primary cue for grouping people in memory. Instead, they appeared as a relatively weak cue that was more evident among participants who showed less categorization by traditional dimensions, a pattern that occurred infrequently in our sample. Together, supporting our hypothesis, these results suggest that social categorization itself is dynamic and interdependent. Sex and to a lesser extent race served as primary organizing dimensions.

Our results supported our first prediction that scar-based categorization is highly malleable, tends to be evident only under narrow and infrequent conditions, and is weaker than categorization based on familiar social-category cues. Our second prediction received little support. Although scar scores were highest when race-based categorization was weak and sex-based categorization was strong, this pattern characterized only a small proportion of participants (16.5%) and did not reflect a general shift toward anomaly-based grouping when traditional cues were weak. When both sex- and race-based categorization were low, scar-based categorization was only slightly above zero, not reliably different from zero, and statistically non-significant. Thus, scars were not a robust organizing cue even when traditional cues were relatively weak. Therefore, our findings suggest that facial anomalies function as a relatively weak and context-dependent social cue: scar-based categorization was negatively associated with the strength of sex- and race-based categorization and did not consistently become a primary basis for grouping even when traditional cues were relatively weak. Because the regression operates on participant-level categorization indices, it does not model conditional cue competition at the level of individual error choices. Accordingly, these results should be interpreted as patterns of association rather than causal suppression among cues.

The error-type distribution shown in Figure 3 also illustrates joint patterns of cue overlap that are not fully captured by the participant-level categorization indices used in the regression analyses. Because these indices summarize overall within-category versus between-category confusions for each cue, they do not directly represent the conditional structure of specific cue combinations at the trial level. As a result, the aggregate scores and the error-type distribution reflect related but not identical aspects of the data. Future work using trial-level choice models may help to more directly examine these conditional dependencies among cues. It is also important to note that the scar categorization score was negative (*M* = −0.17; Figure 4), meaning participants were less likely to confuse individuals who shared scar status than those who differed, indicating that scars did not systematically organize memory despite appearing in certain error combinations shown in Figure 3.

Moreover, because participants’ real-world contact with people who have facial anomalies, as well as age-related differences in such contact, could plausibly influence encoding, we conducted exploratory analyses including exposure frequency, acquaintance type, self-scar status, and age. None of these variables significantly predicted scar-based categorization, indicating that the observed cue-trade-off effects are likely driven by general cognitive mechanisms rather than by individual-difference factors.

The present findings are broadly consistent with prior work on spontaneous categorization. Sex emerged as the strongest and most reliable basis for grouping, consistent with evolutionary accounts, identifying sex as an evolutionarily primary cue that is encoded rapidly and obligatorily (Stangor et al., 1992; Susskind, 2007; Taylor et al., 1978; Kurzban et al., 2001; see Pietraszewski, 2021). Race-based categorization, in contrast, was weak and comparatively unstable, consistent with classic findings from the WSW paradigm that race is not an inherently encoded dimension but instead a flexible cue whose salience depends on context and competing information (Kurzban et al., 2001; Pietraszewski, 2021). Importantly, this modest race effect reflects a well-known feature of the WSW paradigm. By repeatedly exposing faces and requiring discrimination among several individual speakers, the WSW task weakens the interrace categorization asymmetries that underlie the classic other-race effect (Chance et al., 1975) and other-race bias (Lee & Penrod, 2022), the common perception that, when unfamiliar, other-race people all “look alike” (see Flade & Imhoff, 2024). As a result, race differences in categorization tend to be modest and symmetrical in WSW tasks. Therefore, the relatively small race effect we observed is best understood as a genuine categorization pattern shaped by the properties of the WSW paradigm, rather than a reflection of differences in face-recognition accuracy or ingroup identification. Although we did not directly assess whether race-based categorization was symmetrical across racial groups, the magnitude and form of the race effect we observed align closely with the symmetrical categorization patterns typically found in WSW literature.

The present findings further help clarify how evolutionary pathogen-avoidance accounts relate to social categorization. Facial anomalies are often interpreted as cues to infectious disease and can elicit disgust-based avoidance responses in perceivers (Ryan et al., 2012; Klebl et al., 2020). Neural evidence shows that disgust-sensitive responses to anomalous faces predict negative evaluations in anomalous-is-bad bias (Workman et al., 2021; Hartung et al., 2019; Jamrozik et al., 2019), consistent with proposals that anomalies may activate the behavioral immune system (Schaller & Park, 2011). Memory is thought to play an important role in this system: objects or people associated with contamination cues are often remembered better than neutral ones, as shown in work using verbal descriptions of pseudo-contamination (Fernandes et al., 2017), photographs displaying contamination-related cues (Bonin et al., 2019), or videos depicting individuals described as “contagious” (Gretz & Huff, 2019). However, this pattern was challenged by Thiebaut et al. (2022) who found that objects paired with individuals who had scars or burns were not remembered better than those paired with healthy individuals, suggesting that while anomalies may activate avoidance responses, these reactions do not necessarily enhance memory. Aligning with this finding, our results suggest that despite the well-documented anomalous-is-bad stereotype and evidence that anomalous faces elicit pathogen-linked avoidance responses, facial anomalies did not function as a strong or reliable basis for social categorization. Thus, although facial anomalies may evoke negative evaluations due to anomalous-is-bad stereotyping (Paruzel-Czachura et al., 2024), they did not automatically structure memory or guide spontaneous categorization. This distinction reinforces the idea that evaluative bias and perceptual categorization are separable processes: a face can be disliked without becoming a psychologically meaningful category.

The present study employed a small stimulus set, with a single face representing each cell of the 2 × 2 × 2 design. This approach is consistent with many prior Who-Said-What studies, which typically employ a minimal number of faces paired with multiple statements to isolate category structure under controlled conditions (Pietraszewski et al., 2014; Pietraszewski, 2018; Kurzban et al., 2001). Nonetheless, this design limits generalizability and raises the possibility that some effects may partly reflect idiosyncratic properties of individual faces. This concern is especially relevant for the facial anomaly manipulation, for which burn scars were digitally added to faces to be visually salient. Scar appearance was reviewed by the research team for clarity and realism. However, scar realism was not empirically normed or systematically validated. Future research should replicate these findings using larger and more diverse face sets, include multiple exemplars per condition, and independently validate the perceptual realism of facial anomalies. Of note, although statement sentiment was controlled, the semantic content of self-descriptive statements may carry implicit stereotypic associations (e.g., hobbies linked to gender norms). Because each face was paired with a single statement during encoding, such associations could potentially influence memory confusions via statement–face congruence. However, face-statement pairings were randomized across participants, reducing systematic alignment between specific semantic content and social categories at the group level. Future work could further address this possibility by using semantically neutral statements or by independently norming stereotype associations of statement content. Moreover, although our participant-level regression approach is standard in the Who-Said-What literature and addresses our research question directly, alternative analytical approaches could provide complementary insights. For instance, discrete choice models such as conditional logit (Train, 2009) could treat each error trial as a choice among the eight faces, modeling the probability of selecting a given face as a function of which cues it shares with the target while conditioning on the full set of alternatives available on each trial. Such trial-level choice models could examine dependencies among cues at the level of individual error decisions, potentially revealing patterns not captured by aggregate categorization scores.

## 5. Conclusions

The present study shows that we do not treat all visual cues to categorization equally. Sex, as an evolutionarily primary and perceptually privileged dimension, is encoded rapidly and consistently, and emerges as the most stable and robust basis for memory-based grouping. Race, by contrast, was a weak sorting signal. Facial anomalies did not function as an independent or stable basis for grouping. Rather, they operated as low-priority, highly malleable cues whose influence was substantially weaker than that of sex and race. Scar-based categorization statistically appeared only under conditions in which the primary cues, particularly race, provided relatively weaker structure, and such conditions were rare in our data. Therefore, this pattern does not mean that scars form a meaningful social category, nor that anomaly-based grouping is likely to emerge in real-world settings. The absence of scar-based categorization is theoretically important: it indicates that negative evaluations of anomalous faces (anomalous-is-bad stereotyping) do not automatically translate into stronger memory, salience, or categorical organization. In everyday social perception, rich with identity, behavior, emotion, and context cues, facial anomalies are probably not a primary organizing dimension for how most people categorize others in the world.

## Figures and Tables

**Table 1:** Summary Statistics of Categorization Strength for Race, Sex, and Scar Cues.

Cue	*M*	*SD*	*t* (485)	*p*	*d*
Sex	0.29	0.44	14.63	<0.001	0.66
Race	−0.04	0.48	−2.05	0.041	0.09
Scar	−0.17	0.44	−8.43	<0.001	0.38

**Table 2:** Regression model predicting scar-based categorization from race- and sex-based categorization scores and their interaction. Both race- and sex-based categorization negatively predicted scar-based categorization, and their interaction indicates that anomaly-based grouping was lowest among participants who showed strong use of both cues.

Predictor	Estimate (*b*)	*SE*	*t*-Value	*p*
Intercept	−0.11	0.018	−6.07	<0.001
Race	−0.32	0.043	−7.57	<0.001
Sex	−0.15	0.034	−4.42	<0.001
Race × Sex	−0.51	0.062	−8.16	<0.001

**Table 3:** Simple slopes of race-based categorization predicting scar-based categorization at low (−1 SD), mean, and high (+1 SD) levels of sex-based categorization, and tests of slope differences.

		Slope of Race Cue				Slope Difference				
Sex Cue Level	Sex Cue Value	Estimate	*SE*	*t*-Value	*p*	Contrast	Estimate	*SE*	*t*-Ratio	*p*
−1 SD (Low)	−0.146	−0.25	0.05	−5.03	<0.001	Low~Mean	0.22	0.027	8.16	<0.001
Mean	0.289	−0.47	0.03	−14.40	<0.001	Low~High	0.441	0.054	8.16	<0.001
+1 SD (High)	0.724	−0.69	0.03	−20.64	<0.001	Mean~High	0.22	0.027	8.16	<0.001

## Data Availability

Preregistration link: https://doi.org/10.17605/OSF.IO/2SPGF. The materials, data and analysis code are available on Open Science Framework (OSF): https://osf.io/kysne/overview?view_only=5a70a1e22c144cd39340b5ac5d688a74 (accessed on 17 March 2026).

## Supplementary Materials

The following supporting information can be downloaded at: https://www.mdpi.com/article/10.3390/bs16030462/s1, Figure S1. Null distribution of the Sex × Race interaction coefficient. Histogram shows coefficients from 5000 null simulations preserving each participant’s number of errors and the 8-face task structure while randomizing face selection on error trials. The red vertical line indicates the observed interaction (*b* = −0.51), which lies in the extreme tail of the null distribution (*p* = 0.023), indicating that the observed cue competition substantially exceeds what would be expected from shared-error constraints alone. Figure S2. Relationships between scar exposure, acquaintance type, self-scar status, age, and scar-based categorization bias. (A) Acquaintance type and scar bias. Participants who knew someone with a visible scar (coworker or family) showed descriptively lower, but not statistically significant, scar-based bias compared to those without such acquaintances. (B) Self-scar status and scar bias. Participants with visible scars themselves did not differ reliably in bias compared to those without scars. (C) Exposure frequency and scar bias. Frequency of exposure to people with visible scars showed no meaningful linear relationship with scar-based categorization scores. (D) Age × frequency interaction. The association between exposure frequency and scar bias did not vary by age; regression slopes were near zero across the adult lifespan (color gradient represents participant age). Across all panels, higher values indicate greater scar-based categorization bias, and shaded areas show ±1 SE. Table S1. Regression analysis predicting scar-based categorization from race and sex categorization and exposure variables. Table S2. Age moderation analysis. Table S3: Mean scar categorization scores by sex and race cue-profile groups.
