# Supplementary figures and images for: Who Do We Remember? Facial Anomalies, Race, and Sex in Social Categorization
Source: Behav Sci (Basel). 2026 Mar 20;16(3):462. doi: 10.3390/bs16030462 (PMC13023564; doi:10.3390/bs16030462)

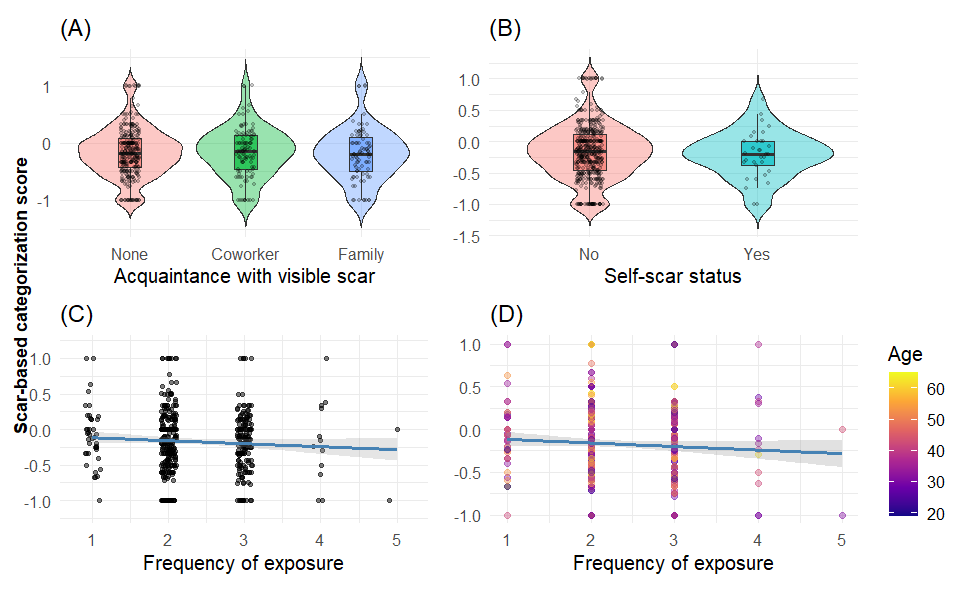

Supplement: Supplementary file 1 [file behavsci-16-00462-s001.zip › Figure_S2.tiff]

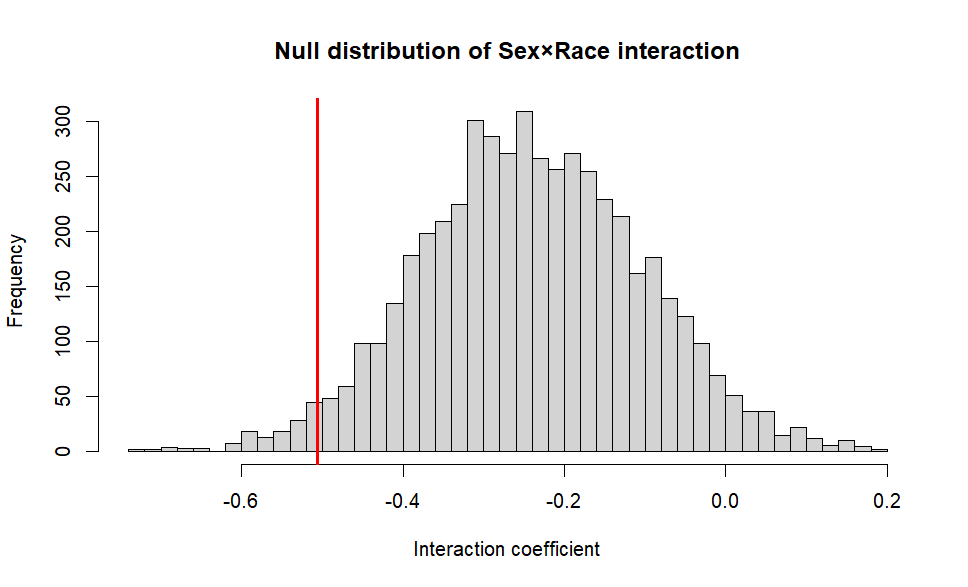

Supplement: Supplementary file 1 [file behavsci-16-00462-s001.zip › Figure_S1.tiff]
